# Use of dietary supplements by pregnant women in Colombia

**DOI:** 10.1186/s12884-018-1758-5

**Published:** 2018-05-02

**Authors:** Robinson Ramírez-Vélez, Jorge Enrique Correa-Bautista, Héctor Reynaldo Triana-Reina, Emilio González-Jiménez, Jacqueline Schmidt-RioValle, Katherine González-Ruíz

**Affiliations:** 1 Centro de Estudios para la Medición de la Actividad Física «CEMA». Escuela de Medicina y Ciencias de la Salud, Universidad del Rosario, Bogotá D.C, Colombia; 2 Grupo GICAEDS. Programa de Cultura Física, Deporte y Recreación, Universidad Santo Tomás, Bogotá, D.C, Colombia; 3 Departamento de Enfermería. Facultad de Ciencias de la Salud, Universidad de Granada, Avda. De la Ilustración, s/n, 18016 Granada, Spain; 4 Departamento de Enfermería. Facultad de Ciencias de la Salud, Universidad de Granada, Grupo CTS-436, Centro de Investigación Mente Cerebro y Comportamiento (CIMCYC), Granada, Spain; 5 Vicerrectoría de Investigaciones, Grupo de Ejercicio Físico y Deportes, Facultad de Salud, Universidad Manuela Beltrán, Bogotá, D.C, Colombia

**Keywords:** Nutrition, Pregnancy, Socio-demographic factors, Prenatal care, Prevalence

## Abstract

**Background:**

During pregnancy, the need for certain nutrients increases. This study assessed the prevalence and socio-demographic factors associated with dietary supplement use in a representative sample of pregnant women in Colombia.

**Method:**

Data for this study were obtained from a cross-sectional, nationally representative survey (ENSIN, 2010). A total of 1856 pregnant women, 13–49 years of age, were recruited. The use of prenatal dietary supplements (Vitamins A, C or E) was treated as a binary outcome (used at some time or never sued during pregnancy when prescribed by a doctor) in multinomial analyses. Sociodemographic data and associated factors were assessed by computer-assisted personal interview technology.

**Results:**

Of the sample, 1123 women (68.6%) reported taking prenatal dietary supplements at some stage during their pregnancy. Most users had a high socioeconomic level (79.5%), were in their third trimester of pregnancy (79.5%), were 30–49 years of age (74.0%), and lived in the central region of Colombia (73.8%). The multivariate logistic regression showed that third trimester of pregnancy (OR 6.2;95% CI 4.0 to 9.3), high educational level (OR 2.3; 95% CI 1.5 to 3.4), high socioeconomic level -SISBEN IV or more- (OR 2.0; 95% CI 1.4 to 2.8), residence in the Atlantic region (north) (OR 2.6; 95% CI 1.7 to 3.6), Eastern region (OR 2.0; 95% CI 1.3 to 3.1), central region (OR 2.6; 95% CI 1.7 to 3.9), Pacific region (west) (OR 1.5; 95% CI 1.0 to 2.3), and belonging to the mestizo (others) ethnic group (OR 1.2; 95% CI 1.0 to 2.6), were all associated with a higher probability of dietary supplement intake.

**Conclusion:**

The prevalence of prenatal dietary supplements in pregnant women in Colombia was found to be substantial. The variables significantly associated with their use were educational level, socioeconomic level, trimester of pregnancy, geographic level and ethnic group. These results indicate the necessity of implementing new health policies that guarantee uniform access to nutritional supplements for all population sectors, especially in countries, such as Colombia, who are currently undergoing a process of nutritional transition.

## Background

Maternal nutrition plays a crucial role in influencing fetal growth and birth outcomes [1]. It is a modifiable risk factor of great importance in public health and is influential in the prevention of adverse birth outcomes, particularly in low-income populations [2]. Deficient dietary intake of vitamins and minerals has been associated with an increased risk of adverse birth outcomes (i.e. miscarriage, low birth weight, preterm birth, and intrauterine growth restriction [IUGR]). Consequently, providing women with vitamin supplements either prior to or in early pregnancy may help prevent pregnancy complications [3].

Adverse birth outcomes are the leading cause of neonatal death among children born without congenital anomalies and often result in short-term and long-term health problems or disabilities, including a potential predisposition to chronic disease in adulthood [1]. Based on evidence from observational studies [4, 5], vitamin supplements, such as folate and B vitamins, are most frequently advocated for the prevention of adverse birth outcomes [6]. In addition, vitamins A, D, E and C, iron, zinc, and selenium are the micronutrients that are generally provided as supplements during pregnancy.

Micronutrient needs increase during pregnancy because of changes in physiology and homeostatic control [7, 8]. The importance of micronutrients has become increasingly apparent, especially in resource-poor settings in which women may enter pregnancy with multiple micronutrient deficiencies [9]. For example, in Colombia 38% of pregnant women have iron deficiency leading to anemia. This is especially prevalent in women of African descent, and those 18–29 years of age [10]. Moreover, 18% of pregnant women have vitamin B12 deficiency or folic acid [11], which can cause neural tube defects, such as spina bifida and anencephaly in the fetus. There is evidence that folic acid supplements during pregnancy decreases the risk of stillbirth, as shown in a comparative study of the use of low and high folic acid supplement dosages among pregnant women in Spain (RR 0.92, 95% CI 0.85 to 0.99, *n* = 79,851 participants) [5]. Although the evidence base is not substantial for antioxidant vitamins, A and C, a Cochrane review found no evidence of differences in early or late miscarriage between women given antioxidant vitamins compared to those in the low antioxidant group (RR 1.12, 95% CI, 0.24 to 5.29, one trial, 110 participants) [6].

Given the risk of malnutrition in low and middle-income countries, it is necessary to measure its prevalence in vulnerable populations, such as children [12], pregnant women [9, 10] and ethnic minorities [13] in order to identify high-risk groups and implement preventive interventions. Despite the fact that malnutrition during pregnancy is a major public health concern, little information is available regarding dietary supplement use in pregnant women throughout the world [14, 15]. Studies conducted in various countries suggest that prenatal dietary supplement use may be less frequent when the mother is younger, belongs to a low-income group, and has a low education level [16, 17]. Currently, in Colombia, public health policies recommend prescribing iron, calcium and folic acid supplements during pregnancy [18].

However, to the best of our knowledge, research has never focused on the potential association between prenatal dietary supplement intake and socio-demographic factors in a nationally representative sample in Latin America. The results obtained could help identify risk groups, and thus provide information to better design interventions that would make the population aware of the need to take dietary supplements to meet nutritional needs.

For this reason, the objective of this study was to evaluate the prevalence and sociodemographic factors associated with dietary supplement intake in a representative sample of pregnant women in Colombia.

## Methods

### Design and participants

In National Nutritional Survey [*Encuesta Nacional de la Situacion Nutricional en Colombia* (ENSIN)], participants were selected through multi-stage stratified sampling to represent 99% of the country’s population [19]. Details of the survey have been published elsewhere [10, 11, 19]. The ENSIN survey included 50,670 households, in which 12,437 women of childbearing age (13–49 years), and 1856 (14.9%) were pregnant (mean age of 24.4 years), completed a food frequency questionnaire. The databases were provided by the Colombian Institute of Family Welfare (in Spanish: Instituto Colombiano de Bienestar Familiar, ICBF), which is responsible for the ENSIN in Colombia. Consent for participation and databases were provided by the ICBF-ENSIN, from the website (https://www.icbf.gov.co/bienestar/nutricion/encuesta-nacional-situacion-nutricional).

All participants and the parents/legal guardians of minors under 18 gave their informed written consent before the study began. The PROFAMILIA Ethics Committee approved the study prior to data collection [*Resolución 8430 de 1993; Ministerio de Salud de Colombia*]. The study was conducted according to the guidelines in the Declaration of Helsinki.

### Data sources

All information collected was obtained through face-to-face interviews conducted at each site. The interview covered different aspects of pregnancy, including general information, sociodemographic variables, and dietary information. Use of prenatal micronutrients (such as Vitamin A and/or Vitamin C and/or Vitamin E) was treated as a binary outcome (at some time used or never used in pregnancy though always with a doctor’s prescription) in all analyses.

The following sociodemographic variables were defined as associated factors: (i) age (13–17, 18–29, or 30–49 years); (ii) pregnancy trimester (first, second, or third); (iii) education level (elementary and/or secondary, university and/or postgraduate); (iv) urbanicity (urban or rural); (v) ethnicity.

Ethnicity was divided into the following subgroups: (a) indigenous (indigenous ethnicity included 1,378,884 [3.4%] people belonging to various groups, i.e. Emberá, Mokane, Ika, Kankuamo, Awuá, Misak, Nasa and Wayuu, [20]; (b) Black or Afro-Colombian; (c) others (e.g. mestizo). Mestizo Colombians have mixed European and Amerindian ancestry. They are the largest ethnic group in the country (49%–58% of the population).

Geographic region was divided into the following areas: (a) Atlantic (North); (b) Eastern; (c) Central; (d) Pacific (West); (e) Bogota; (f) National Territories comprising the departments of Putumayo, Amazonas, Arauca, Casanare, Guainía, Vichada, and Vaupés in the southern part of Colombia.

The socioeconomic status was determined according to the national SISBEN index (System of Identifying Potential Beneficiaries of Social Programs; SISBEN, initials for the Spanish) [10, 11, 19]. The SISBEN is a system designed by the Colombian Government to identify families who would benefit from social programs. Based on this information, six levels are defined with one being the poorest and six being the wealthiest. Households with SISBEN levels of I or II are regarded as the most vulnerable and are targeted in social programs. In contrast, a SISBEN level IV, (including levels IV–VI) is considered to be the least vulnerable sector of society [10, 11, 19]. For the purposes of this study, SISBEN scores were classified in four categories (I, II, III, and IV or higher) to improve the efficiency of the analyses.

### Data analysis

Univariate tests were used to summarize and identify patterns in the data with a focus on the selected variables (Table 1). To investigate an association between prenatal supplement intake and multiple predictor variables (age, pregnancy trimester, education level, urbanicity, geographic region, ethnicity, and SISBEN socioeconomic level) we used a multilevel logistic regression model to estimate the magnitude of association in the form of odds ratios (ORs) and 95% confidence intervals (CIs), between prenatal supplement intake and selected predictor variables. Additionally, we fitted two models. First, we included the gestational variables in the model (model 1: adjusted by age and trimester of pregnancy) to investigate the extent to which prenatal supplement intake differences were explained by the individual composition of the women. Second, we included the sociodemographic factors variables (model 2: education level, geographic area, socioeconomic level, and urbanicity). This was in addition to the variables already included in model 1 to discover whether this prenatal dietary supplement use was conditioned by specific sociodemographic characteristics. Variables with *P* < 0.10 were considered for the multilevel logistic regression analysis. All analyses were performed with the use of the complex survey design routines of the SPSS Statistical software package version 20 (SPSS, Chicago, IL, USA).

**Table 1:** Prevalence and socio-demographic factors associated with prenatal supplement intake in a representative sample of pregnant women in Colombia

Characteristics	Prenatal supplement intake			
	Yes		No	
	n (%*)	95% CI**	n (%*)	95% CI**
Total (*n* = 1865)	1123 (68.6)	(67.2–69.8)	742 (31.4)	(29.7–33.0)
Age (years)				
13 to 17 (*n* = 249)	142 (65.6)	(62.0–68.2)	107 (34.4)	(30.0–37.5)
18 to 29 (*n* = 1204)	727 (67.4)	(65.8–68.7)	477 (32.6)	(30.6–34.5)
30 to 49 (*n* = 412)	254 (74.0)	(71.6–75.8)	158 (26.0)	(22.9–28.5)
Trimester of pregnancy				
First (*n* = 292)	96 (41.3)	(36.6–44.8)	196 (58.7)	(55.8–60.8)
Second (*n* = 978)	602 (69.3)	(67.7–70.6)	376 (30.7)	(28.5–32.7)
Third (*n* = 595)	425 (79.6)	(78.1–80.8)	170 (20.4)	(17.8–22.6)
Education level				
Elementary or secondary (*n* = 1537)	864 (64.6)	(63.0–66.0)	673 (35.4)	(33.6–37.0)
University or postgraduate (*n* = 328)	259 (82.0)	(80.6–83.1)	69 (18.0)	(14.0–21.0)
Socioeconomic level				
Level I (*n* = 1088)	561 (61.2)	(59.2–62.9)	527 (38.8)	(36.8–40.6)
Level II ^a^ (*n* = 176)	106 (62.3)	(58.3–65.0)	70 (37.7)	(32.1–41.6)
Level III ^a^ (*n* = 136)	98 (77.1)	(73.6–79.4)	38 (22.9)	(17.2–26.6)
Level IV or more (*n* = 465)	358 (79.5)	(78.1–80.6)	107 (20.5)	(17.5–23.0)
Geographic area				
Atlantic (North) (*n* = 438)	302 (71.7)	(69.8–73.3)	136 (28.3)	(25.6–30.5)
Eastern (*n* = 258)	179 (69.2)	(66.9–71.0)	79 (30.8)	(26.7–33.9)
Central (*n* = 381)	264 (73.8)	(71.5–75.5)	117 (26.2)	(23.2–28.6)
Pacific (West) (*n* = 246)	128 (59.0)	(55.6–61.6)	118 (41.0)	(37.8–43.5)
Bogotá ^a^ (*n* = 98)	68 (69.4)	(65.9–71.8)	30 (30.6)	(24.4–34.7)
National territories (South) (*n* = 444)	182 (49.8)	(46.3–52.6)	262 (50.2)	(47.0–52.7)
Urbanicity				
Urban (*n* = 1186)	804 (71.0)	(69.8–72.2)	382 (29.0)	(26.8–30.9)
Rural (*n* = 679)	319 (47.0)	(58.9–64.1)	360 (53.0)	(36.2–39.8)
Ethnic group^≠^				
Indigenous (*n* = 272)	95 (50.2)	(45.1–53.6)	177 (49.8)	(44.1–53.6)
Black *or* Afro-Colombian (*n* = 202)	109 (63.6)	(59.4–66-6)	93 (36.4)	(33.2–38.6)
Others (*n* = 1380)	908 (70.3)	(69.0–71.5)	472 (29.7)	(27.8–31.4)

*All women analyzed by ethnic group were *n* = 1854. Another two belonged to “Raizal del archipiélago”, but they were not analyzed because this group did not have a representative sample

**It is not correct to calculate the percentages from the “n” in this table. These calculations were taken from the weight of the values ​​given to each subject

^≠^data from 1854 participants

^a^ Since the coefficient of variation is more than 20% of the deficiency prevalence, the variation should be interpreted with caution

## Results

There were 1123 women ([68.6%] 95% CI, 67.2% to 69.8%) who reported taking prenatal dietary supplements at some stage during their pregnancy. Factors associated with a higher intake of dietary supplements were the following: (i) university or postgraduate education (82%); (ii) third trimester of pregnancy (79.6%); (iii) high socioeconomic level, SISBEN IV or higher (79.5%); (iv) age of 30–49 years (74%); (v) residence in a central region (73.8%), and Atlantic region (71.7%) of Colombia. In addition, pregnant women living in urban areas (71.0%) and with an ethnicity of others (mestizo) also had a high prevalence of dietary supplement use (Table 1).

**Fig. 1:**
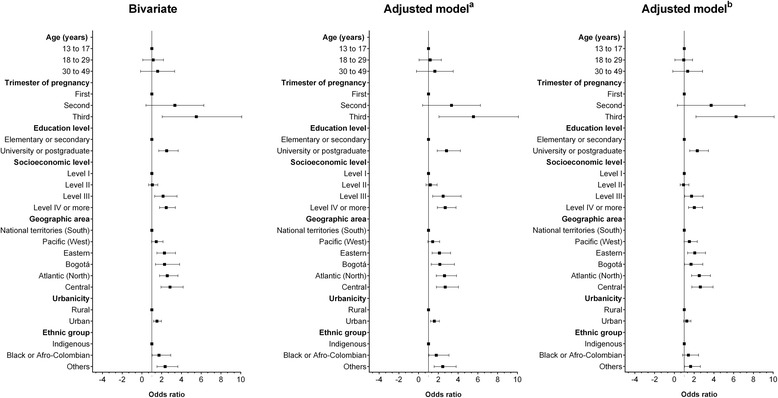
Socio-demographic factors associated with prenatal supplement intake in a representative sample of pregnant women in Colombia. Data in odds ratios (95% confidence interval) ^a^ adjusted by age and trimester of pregnancy. ^b^ adjusted by age, trimester of pregnancy, education level, geographic area, socioeconomic level, and urbanicity

Figure 1 shows the results of the logistic regression analysis. Once the adjustment was performed (by age, pregnancy trimester, education level, geographic area, socioeconomic status, and urbanicity), a higher probability of prenatal supplement use was found to be associated with the following: (i) third trimester of pregnancy (OR 6.2;95% CI 4.0 to 9.3), (ii) high educational level (OR 2.3; 95% CI 1.5 to 3.4), (iii) high SISBEN IV or higher SES (OR 2.0; 95% CI 1.4 to 2.8), (iv) residence in the Atlantic region (north) (OR 2.6; 95% CI 1.7 to 3.6), (v) Eastern region (OR 2.0; 95% CI 1.3 to 3.1), (vi) central region (OR 2.6; 95% CI 1.7 to 3.9), (vii) Pacific region (west) (OR 1.5; 95% CI 1.0 to 2.3), and (viii) being part of the mestizo (others) ethnic group (OR 1.2; 95% CI 1.0 to 2.6).

## Discussion

The results of this study show that a high percentage of women (68.6%) affirmed that they had taken dietary supplements at some time during their pregnancy. This was particularly the case for women, who had a high socioeconomic status and education level, who lived in the central, pacific (west) or Atlantic (north) regions of Colombia, and who were in the third trimester of their pregnancy. Our results differ from those obtained by Titlayo et al. [21] in their study of pregnant women in Malawi, where only 37% of the subjects reported taking dietary supplements during their pregnancy. However, despite the generally lower consumption of these supplements among pregnant women in Malawi, factors that influenced their supplement intake during pregnancy included the participants’ residence in an urban area as well as a high education and socioeconomic level. These coincided with the significant factors highlighted in our study and which reflect unequal access to these dietary supplements. In other words, pregnant women who would most benefit from taking these supplements are those that have fewer possibilities of obtaining them.

In this same line, Taye et al. [22] conducted a study of 634 Ethiopian women and observed that pregnant women with a high education level and socioeconomic status were more likely to adhere and comply with the regular use of dietary supplements. They probably had a better knowledge of and easier access to information regarding the importance and benefits derived from taking these supplements during pregnancy [23]. Furthermore, the reasons that could justify differences in supplement intake among pregnant women in rural and urban areas could be related to the inequality of opportunities, which is characteristic of low and middle-income countries such as Colombia. For this reason, women in urban areas doubtlessly have better access to health services than women in rural areas [21]. Therefore, in our study, women who had a low socioeconomic status and education level, were less likely to adhere and comply with the regular use of dietary supplements during pregnancy.

Previous studies also reported that pregnant women living in urban areas and in geographic regions with a higher level of structural and economic development show a greater adherence to health programs that include the prolonged intake of vitamin supplements during pregnancy [24, 25]. Moreover, ethnic group was another significant factor in relation to the use of dietary supplements by pregnant women in Colombia. In this regard, mestizo women maintained a more consistent pattern of vitamin intake. Other studies [26–28], show greater deficiencies in dietary supplementation in pregnant women belonging to ethnic minorities. In countries like Colombia where economic constraints place limits on achieving dietary adequacy, food-based interventions are one way of improving the nutritional status of vulnerable populations such as pregnant women.

As can be observed in the results of the adjusted regression analysis, pregnant women were found to be more likely to take prenatal dietary supplements if they were in the third trimester of pregnancy, had a higher education level, and resided in the Atlantic regions in north Colombia, pacific (west) region or in the central region of the country. These results are clinically and socially relevant for Colombia since, according to WHO [29], each pregnant woman should receive dietary supplements (i.e. vitamins and minerals) one month before conception until the end of the first pregnancy trimester though the current tendency is to prolong intake during the entire pregnancy.

However, the results obtained show that there was a greater probability of receiving supplements in the third trimester of pregnancy. In contrast, there was no guarantee of receiving essential vitamins and minerals in the other two trimesters of pregnancy [30]. This situation is troubling, especially if we consider that 18% of pregnant women in Colombia have anemia and 37% suffer from iron deficiency [31]. These results indicate the necessity of implementing new health policies that guarantee uniform access to nutritional supplements for all population sectors [32].

Various studies have found that poor nutritional status at birth is associated with physical and cognitive impairments during infancy and childhood that may persist into adulthood [33–35]. Research on animals and epidemiological studies indicate that metabolic adaptations in suboptimal intrauterine environments are associated with the onset of cardiovascular disease, diabetes, and other chronic illnesses later in life [36]. These changes, referred to as fetal programming, underpin the “fetal origins of adult disease” hypothesis [37].

The results of our study highlight the fact that Colombia must implement interventions and/or reproductive health programs with a view to guaranteeing an all-encompassing obstetric care system. This includes meeting nutritional needs throughout the entire pregnancy, regardless of the education level or geographic location of the pregnant woman [38]. An effective strategy would thus be to follow the recommendations of the *Grupo Asesor en Micronutrientes* [Micronutrients Advisory Group] of MERCOSUR [39], which include a varied diet, iron supplements for pregnant women and children under two, and food fortification (i.e.: wheat flour, maize flour, and rice). Another important initiative is the program for the prevention and reduction of nutritional anemia devised and fostered by the President of Colombia, the Colombian Ministry of Health, and the United Nations World Food Program. The implementation of these measures would help to combat nutritional deficiencies in the general population, and especially in pregnant women independent of the education level and socioeconomic status.

The Colombian Ministry of Health has acknowledged the need to improve the nutritional status of women during pregnancy. Priorities of the *Decanal National Public Health Strategy*: 2012–2021 [40] include the “reduction of protein energy malnutrition and micronutrient deficiencies in women and improving care for pregnant women, including extra dietary intake and rest for increased weight gain during pregnancy”. Key Colombia Ministry of Health recommendations for women during pregnancy are the following: (i) total weight gain of at least 7 kg; (ii) consumption of one extra daily meal (unspecified); (iii) a course of 90 iron-folic acid tablets (provided at a prenatal care center); and (iv) a minimum of four prenatal care visits [41]. All these recommendations would be an important step towards improving the health of pregnant women and of unborn children. It would also help to reduce unequal access to nutritional resources and supplements.

Consequently, lessons learned from the Colombian context through analyses of available surveillance data as well as from the future process and impact evaluation of policies and programs have the potential to inform malnutrition prevention efforts in other settings. In Colombia, the following three strategies can be implemented to improve nutritional status: (i) dietary interventions that promote increased consumption of a greater variety of locally available nutritious foods; (ii) fortification of commonly consumed foods/seasonings; (iii) provision of nutritional supplements that help individuals achieve required intakes of certain nutrients often lacking in staple diets. For this reason, it is essential to educate health personnel about nutrition and nutritional needs. On a positive note, coordination with other educational and health and wellness strategies has produced viable and sustainable interventions [11]. This practice supports the Millennium Development Goals [42] to promote the health of mothers and infants in low and middle-income countries.

Our study has certain limitations. The most substantial limitation of this study was the source of denominator data. We examined the effect of the associated factors and made use of the best data available to estimate the population at risk for each age group. The distributions of women, 13–49 years of age, who were surveyed in the 2010 ENSIN data set, were applied to the 2009 population projections in Colombia. Given that the 2010 ENSIN was reported data, the distribution of the socio-demographic variables used to obtain the population at risk in our study was limited by the quality of the survey results. Difficulties in estimating risk-factor group-specific denominator data for less populated regions also limited our ability to assess the joint effect of education and insurance in these data. Another limitation was the cross-sectional design that does not permit the establishment of causality.

One of the evident strongpoints of our study is the fact that it is the first one to assess the prevalence and socio-demographic factors associated with dietary supplement intake in Colombia in women, 13–49 years of age. For this purpose, multimodal regressions were used to control for confounding multiple socio–demographic variables and for missing values. Another strongpoint of this study is the size of the sample population, which can be used to make comparisons in subsequent studies. Furthermore, this study helps to remedy the lack of available information in Latin America with regard to the use of dietary supplements by pregnant women. In this regard, the results obtained are original as well as clinically and epidemiologically important.

## Conclusions

The research results presented in this paper showed that during prenatal care in Colombia, there is a substantial use of dietary supplements in pregnant women with a high socioeconomic status and education level. Moreover, the high prevalence of supplement intake during pregnancy is significantly associated with factors such as high education level, third trimester of pregnancy, ethnic group, and geographic location. Our findings point to the need to further evaluate, foster, and enhance undernutrition prevention, diagnosis, treatment, and follow-up. Furthermore, these results indicate the necessity of implementing new health policies that guarantee uniform access to nutritional supplements for all population sectors, especially in countries, such as Colombia, who are currently undergoing a process of nutritional transition.

## Abbreviations

ENSIN: 2000 Colombian National Nutrition Survey (initials for the Spanish)
IUGR: Intrauterine growth restriction
PROFAMILIA: Asociación Pro-bienestar de la Familia Colombiana (in Spanish)
SISBEN: System of Identifying Potential Beneficiaries of Social Programs (initials for the Spanish
